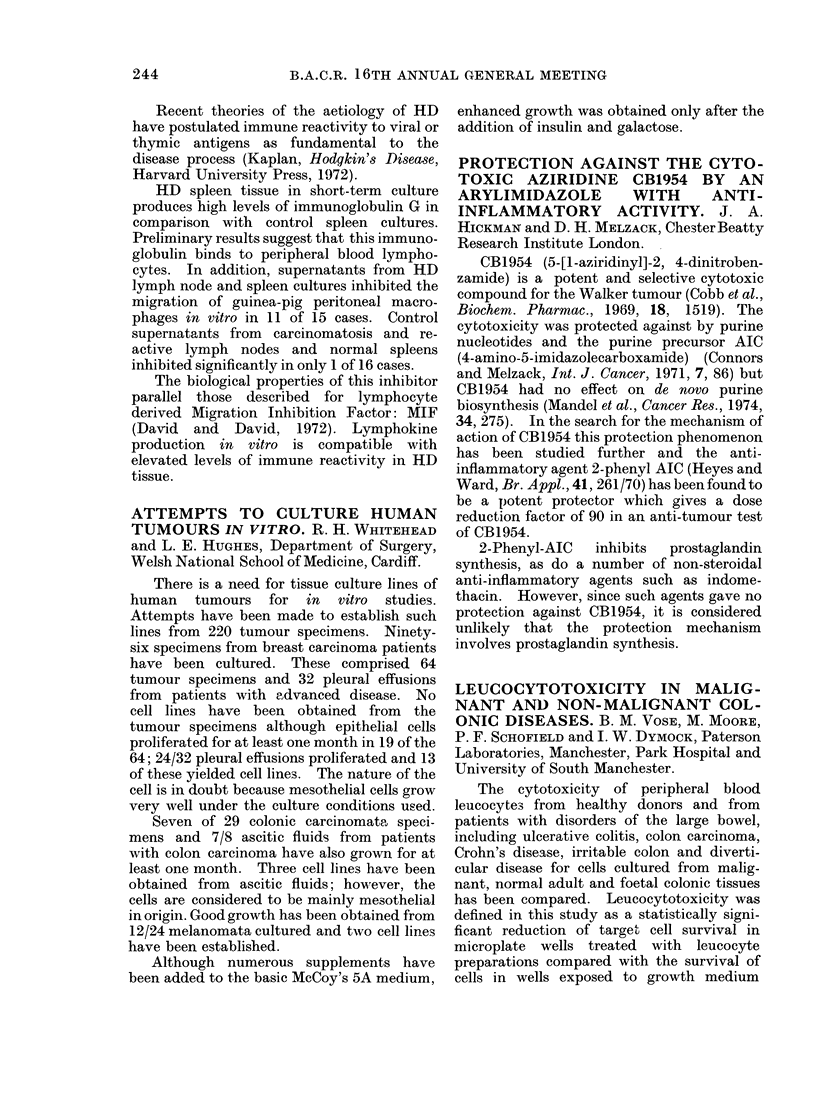# Proceedings: Attempts to culture human tumours in vitro.

**DOI:** 10.1038/bjc.1975.168

**Published:** 1975-08

**Authors:** R. H. Whitehead, L. E. Hughes


**ATTEMPTS TO CULTURE HUMAN TUMOURS *IN VITRO*.** R. H. WHITEHEAD and L. E. HUGHES, Department of Surgery, Welsh National School of Medicine, Cardiff.

There is a need for tissue culture lines of human tumours for *in vitro* studies. Attempts have been made to establish such lines from 220 tumour specimens. Ninety-six specimens from breast carcinoma patients have been cultured. These comprised 64 tumour specimens and 32 pleural effusions from patients with advanced disease. No cell lines have been obtained from the tumour specimens although epithelial cells proliferated for at least one month in 19 of the 64; 24/32 pleural effusions proliferated and 13 of these yielded cell lines. The nature of the cell is in doubt because mesothelial cells grow very well under the culture conditions used.

Seven of 29 colonic carcinomata specimens and 7/8 ascitic fluids from patients with colon carcinoma have also grown for at least one month. Three cell lines have been obtained from ascitic fluids; however, the cells are considered to be mainly mesothelial in origin. Good growth has been obtained from 12/24 melanomata cultured and two cell lines have been established.

Although numerous supplements have been added to the basic McCoy's 5A medium, enhanced growth was obtained only after the addition of insulin and galactose.